# Challenges of anticipation of future decisions in dementia and dementia research

**DOI:** 10.1007/s40656-022-00541-8

**Published:** 2022-11-14

**Authors:** Julia Perry

**Affiliations:** Department of Medical Ethics and History of Medicine, University Medical Center Göttingen, Humboldtallee 36, 37073 Göttingen, Germany

**Keywords:** Anticipation, Dementia, Research participation, Advance research directives, Future

## Abstract

Anticipation of future decisions can be important for individuals at risk for diseases to maintain autonomy over time. For future treatment and care decisions, advance care planning is accepted as a useful anticipation tool. As research with persons with dementia seems imperative to develop disease-modifying interventions, and with changing regulations regarding research participation in Germany, advance research directives (ARDs) are considered a solution to include persons with dementia in research in an ethically sound manner. However, little is known about what affected people deem anticipatable.

This contribution provides a critical reflection of the literature on anticipation and of a qualitative study on the assessment of ARDs with persons with cognitive impairment in Germany. It combines theoretical and empirical reflections to inform the ethical-legal discourse.

Anticipation involves the conceptual separation of the past, the present, and the future. Including dimensions such as *preparedness*, *injunction*, and *optimization* helps in establishing a framework for anticipatory decision-making. While dementia may offer a window of time to consider future decisions, individual beliefs about dementia including fears about stigma, loss of personhood, and solitude strongly impact anticipating sentiments. Concepts of anticipation can be useful for the examination of uncertainty, changing values, needs, and preferences interconnected with the dementia trajectory and can serve as a means to make an uncertain future more concrete. However, fears of losing one’s autonomy in the process of dementia also apply to possibilities of anticipation as these require cognitive assessment and reassessment of an imagined future with dementia.

## Introduction

An advance research directive (ARD) is a recently amended legal tool for competent people to express their willingness or objection to participation in clinical research, in advance, for the time they are not competent to make their own decisions (Jongsma et al., 2020). In Germany, ARDs have recently become a necessary condition for the previously prohibited participation of incapacitated people in research with group-benefit with the amendments in the Medicinal Products Act (Deutscher Bundestag, 2016). In light of these legal changes and the growing ethical interest in ARDs for dementia research (Heinrichs, 2021; Götzelmann et al., 2021; Werner & Schicktanz, 2018; Davis, 2017; Jongsma & van de Vathorst, 2015), I argue for the need to reflect on the fact that anticipatory decisions become relevant in this context. Anticipation as such is an important aspect in the context of dementia which, however, has rarely been discussed regarding planning ahead in the field of bioethics.

That anticipation can be considered an important aspect of human life, as a means to navigate through everyday life, and thus has an everyday meaning, is often overlooked. According to the words by the scientist Alan Kay *the best way to predict the future is to invent it*, anticipation differs from seeing the future as pure fate that one is simply at the mercy of. Hence, anticipation can be regarded as a temporal alignment with which the future can be made tangible (Lemos Dekker, 2020). Conceptions of the future can inform individual as well as collective action in the present, ways of knowing, and predicting (Kozubaev et al., 2020; Granjou et al., 2017). In the context of a chronic, progressive, and late-onset condition such as dementia, in which individuals are confronted with future loss of agency and perceived decision-making capacity, anticipation may particularly serve as a means to make an uncertain future more concrete.

Anticipatory decisions regarding future research participation hinge on two distinct forms of anticipation – anticipation towards one’s dementia trajectory and anticipation towards one’s willingness to participate in research. For both forms of anticipation, this can include the consideration of moral uncertainty such as potential changes in one’s identity, one’s values, preferences, needs, and concerns as well as psychological uncertainty. For persons with (a high risk for) dementia, this could more concretely include the uncertainty regarding the non-linear development of the condition and the difficulty to imagine how one may feel with increased loss of cognitive abilities. Temporal uncertainty has a significant effect on future biographies – of those directly affected as well as family members. Dementia can be considered an unpredictable illness trajectory (Sikes & Hall, 2018), which could involve feelings of anxiety regarding a potentially hopeless vision of the future (Swallow, 2017). Feelings of uncertainty may be substantiated by public policy’s current focus on research and the continuous search for potentially effective treatments. Regarding research participation, this more concretely could entail entanglements of hope and uncertainty: on the one hand, the willingness to participate in research to contribute to potential medical advances as well as to research in the life sciences and, on the other hand, the fear of changing preferences and values in the course of dementia and the fear of not being able to express these anymore, potentially combined with reliance on other people to potentially make decisions in such a case (Bethell et al., 2018). This prospect is strongly associated with being vulnerable and unsure about the ability to, for example, withdraw from research. Research participation of people who do not have full decisional capacity is regarded as highly controversial. One specific reason was the so-called *slippery slope argument* referring to the historical abuse of trust and human dignity during the Nazi regime, under which psychiatric patients were systematically discriminated against, experimented with, and killed. Such concerns are still prominent in societal and expert debates about medical research practice, not only in Germany, but internationally (Strous, 2007; Helmchen & Lauter, 1995).

In medical ethics in general, enabling autonomy to make healthcare decisions over time is a central aim (Boenink, 2010). In the case of dementia, however, this ability may be or become impaired in its course. Research ethics guidelines largely encourage supported or shared decision-making and may require researchers to seek consent from proxies when involving people in dementia research (Thorogood et al., 2018). For consent, the decision-making individual must be provided with sufficient information to make an informed decision, whether regarding treatment or research (Rehbock, 2013). Drawing the line between competence and non-competence is highly debatable in the field of medical ethics, especially concerning slowly progressing conditions. Regarding decisions for or against research participation in the context of dementia, the question arises how the declaration of one’s will should be assessed, given the inherent liminality of the condition (Birt et al., 2017; Rehbock, 2013). With the legal amendment, Germany is now one of the few countries that very concretely has to deal with the tool of ARDs. The legal debate regarding ARDs in the recent years has predominantly focused on the ethical acceptability under the aspect of the instability of an individual’s preferences and values or on the ethical justification based on concepts of autonomy. From an empirical-ethical perspective, the ethical debate has focused on whether affected individuals are willing to draft and actually use an ARD (Heinrichs, 2021; Götzelmann et al., 2021; Jongsma et al., 2020). However, what is missing in the current debate is a focus on what the concrete challenges are for drafting an ARD, namely referring to the anticipatory aspect of ARDs. What is deemed anticipatable in the context of dementia and related research thus should be examined more closely from an ethical and sociological-empirical perspective to better understand the practicability and potential obstacles for drafting ARDs.

Therefore, this article focuses on potential challenges of anticipation for drafting an ARD and for advance care planning (ACP) in the context of dementia. The leading research question is: How is the practice of anticipation challenged in the context of dementia and dementia research? To address this question, my contribution has the following argumentative structure: I first consider origins and selected conceptual developments of anticipation in the field of sociology for the Background section. Here I consider the five key dimensions of anticipation identified by Adams et al. (2009) which provide a fruitful starting point to assess conceptions of anticipation relevant in the context of dementia and dementia research. I have selected this framework to provide a theoretically informed reflection on various forms of anticipation which can enhance the practical ethical debate on advance planning in a biomedical context. In a second step, I provide a brief historical overview of advance planning in medical and research contexts as well as of relevant historical events shaping the current practice of research including people with decreased decisional capacities. This part underlines how advance planning should be seen as a form of anticipation that needs further analysis regarding its epistemic, social, and normative challenges. To address concrete conceptions of anticipation in practice, it becomes necessary to move beyond theoretical assumptions and discussions in medical ethics and sociology as well as legal requirements and delve into concrete lived experience of affected people. Thus, in a third step, in the main part, I provide an analysis that is empirically informed by interviews conducted with affected people in Germany in early stages of dementia or with cognitive impairment^1^. This empirical study addresses a research gap, as affected people’s anticipation of dementia and especially their anticipation towards future research participation have rarely been examined. The recent amendments in the Medicinal Products Act, making the drafting of an ARD a necessary condition to participate in research with group-benefit for people with decisional incapacity, encourages such an examination. Relevant insights into practical experiences can be drawn from empirical ethics with the aim of integrating a normative reflection of the empirical material (Huxtable & Ives, 2019). As qualitative approaches necessitate pertinent theoretical integration (Stam, 2000), theoretical discussions will, in turn, also benefit from the integration of empirical data, especially when referring to a topic which is currently being explored and is in need of development for clinical implementation. Integrating the perspectives of those affected can contribute to the practice-oriented assessment of such theoretical assumptions. In this part, specific aspects of dementia and related perceptions of time and liminality inherent to dementia are discussed. For the empirical analysis, I deductively use Adams et al.’s framework to identify relevant concepts and practices of anticipation from an everyday life perspective. The analysis is guided by five dimensions described conceptually by Adams et al. (2009). Additionally, I discuss strengths and weaknesses of this approach and inferences that can be drawn from affected people’s assessment to inform bioethical discussions on anticipation. This is where this contribution provides preliminary insights by combining theoretical concepts and deductively exploring their empirical relevance. Finally, I point briefly to avenues of future research as well as to practical implications of the practice of anticipation in the context of dementia and dementia research.

## Background

In the following Background section, I briefly introduce the two main fields which are both relevant for my interdisciplinary analysis. First, I introduce the concept of anticipation as mainly discussed in the field of sociology, specifically the sociology of future. Second, I introduce the reader to historical and conceptual debates on dementia.

### Anticipation and health-related decisions

#### The concept of anticipation and the sociology of future

Anticipation is defined as the action of anticipating something, i.e. an expectation or prediction. Anticipation accordingly requires a temporal alignment, an orientation towards the future and can become apparent in numerous areas of everyday life. However, different notions of anticipation exist. While the discipline of sociology predominantly considers the present and the past (Beckert & Suckert, 2021), as early as 1916, the sociologist Edward Alsworth Ross described the principle of anticipation as affecting individuals favorably or unfavorably resulting in modifying behavior or creating a possibility for action. As put forward by Tavory (2018), any form of human action or agency necessitates thoughts about moving forward in time. First, anticipatory expectations or future orientations can be considered an integral part of human agency as well as meaning-making and have a highly individualistic component (Tavory, 2018; Adams et al., 2009; Giddens, 1991, pp. 14, 175). Second, engaging with the future can be considered a measure of taking responsibility for what is yet to come, as commonly used for the responsibilization of technological development (Selin, 2008; Schicktanz & Schweda, 2012; Beck, 1992, p. 58). Third, anticipation can be considered a performative role in which individuals act on expectations (Selin, 2008; Goffman, 1959, pp. 24–26). Anticipation can be further regarded as a temporal alignment with which future events and the future as a whole can be made tangible (Lemos Dekker, 2020). Conceptions of the future can inform individual as well as collective action in the present, ways of knowing, and forecasting, as such anticipatory practices can be regarded as forms of risk management by (re)envisioning futures and exploring different alternatives (Kozubaev et al., 2020; Beck 1992, pp. 23–24; Giddens 1991, pp. 18, 82). Anticipation can be considered a process of “establishing, collapsing, and renegotiating the temporal distance between present and future, bringing the future into the present while also, and simultaneously, keeping the future at bay as a continuous ‘not yet’” (Lemos Dekker, 2020, p. 1).

Relevant authors in the field of sociology and specifically future-oriented visions, such as Beck’s concepts of environmental catastrophes, as the basis of risk society (1992, p. 58), and Giddens’s concepts of reflexivity and future-orientation, based on the need to manage and identify risks, directed at action of human agency (1994, pp. 184–197) have sustainably shaped the focus on perceptions of the future in the field of sociology. This includes social science studies of uncertainty, risk, prediction, prevention, precarity, contingency, vulnerability, hope, aspiration, imaginaries, planning, and responsibility, to name a few (e.g., Beckert & Suckert, 2021; Stephan & Flaherty, 2019). However, as opposed to the sociology of time, comprehensive literature on ‘future sociology’ or ‘sociology of the future’, especially focusing on epistemological perspectives, can still be considered an emerging field (Beckert & Suckert, 2021). Considerations of time and future perspectives can both contribute to the relevance of life experiences. Thus, approaches of the sociology of futures^2^ and anticipation can be regarded as useful frameworks for exploring individuals’ dealing with future events and managing uncertainties as well as for exploring collective scenario planning. A dominant field includes current climate change debates, where political or scientific approaches are often based on the practice of foresight and intervention (Granjou et al., 2017; Swallow, 2017; Selin, 2008; Giddens, 1993, pp. 128–129). Brown and Michael (2003) contend that representations of the future can be helpful but also are highly unreliable.

In this article, I take an epistemological approach to perceptions of the future based on individual values, desires, and expectations. Here, I refer to anticipation as the management of time as a resource and focus on how the future plays an inherent role as a conceptual possibility (Adams et al., 2009)^3^. The future can be understood or anticipated in numerous ways. Assumptions about the future can be formulated, revised, or changed completely, while the underlying aim of seizing certainty remains the same (Adams et al., 2009). Valuing anticipation as meaningful may vary among individuals; however, anticipation, in general, can be considered the tangible outcome of a speculative future led by concrete actions taken in the present (Chiffi et al., 2020).

Adams et al. (2009) frame anticipation, in particular, as an affective state driven by predictable uncertainty. The underlying motivation to anticipate can stem from a multitude of emotions ranging from fear or worry to the urge to know. In times of social transformations or also in the context of individual changes in life, it may be perceived as especially meaningful (Adams et al., 2009). Anticipation as an affective state thus can be considered more than a mere reaction and rather a means of “actively orienting oneself temporally” (Adams et al., 2009, p. 247). With anticipation, “the future sets the conditions of possibility for action in the present […], through anticipation the future arrives as already formed in the present” (Adams et al., 2009, p. 249).

The concept of inhabiting time or the notion of temporality as such is not new. In technology development, biomedicine, environmental sciences as in politics, the optimization of future applications or policies has for a long time included the notion of future optimization (Rose, 2007, pp. 17–18, 20, 82, 262; Bijker et al., 1987). This entails the active involvement with prediction, imagination, and anticipation of potential future outcomes, often justifying current efforts in favor of a *better* future (Adams et al., 2009; Selin, 2008). In philosophy, more abstract attempts of utopian vs. dystopian anticipatory thinking (Bloch, 1985 (*Das Prinzip Hoffnung*); Jonas, 2003 (*Das Prinzip Verantwortung*)) have shaped bioethical debates until today.

In the aforementioned fields, anticipation can be considered rather on the collective level, i.e. anticipating for the good of the people, whereas anticipation in other fields, such as individual decisions in the biomedical context can be considered rather on the individual level. Different forms of anticipation are guided by social and cultural norms and, conversely, acts of anticipation are ascribed certain social and cultural norms. Further, the present is contingent on the past, for example, by means of lived experience in which past experience is utilized for making future projections, distinguishing anticipation from mere speculation (Adams et al., 2009). Such lived experience, as described in phenomenological research, can reveal relevant structures, meanings, and values of individuals (Rich et al., 2013). Accessing individual conceptions and values becomes highly relevant for documenting future health-related decisions. For considering the introduction and people’s assessment of ARDs, I regard the concept of “pragmatic subjectivism” as productive in this context. The concept of pragmatic subjectivism, as described by Haybron and Tiberius (2015), puts forward that certain policies can only be justified if based on the values of those affected with the underlying aim of improving their well-being. Specifically, pragmatic subjectivism is based on the understanding that certain policies should improve the beneficiary’s life in accordance with his or her standards, thus the concept aims at integrating what matters to an individual’s well-being. This approach places importance on the promotion of well-being as the respective individuals perceive it, which necessitates the focus on values that form individuals’ conceptions of well-being (Haybron & Tiberius, 2015). For making health-related decisions the concept of well-being is central, making the concept of pragmatic subjectivism very applicable to the healthcare context. Depictions of well-being should help in understanding the subjective experience of life. For this, appropriate frameworks are necessary in healthcare policy that consider individual values promoting well-being and the broad range of values within society (Hall, 2016). According to Hall (2016) an important aspect of subjective experience entails the notion of being mistaken about what was originally considered important or unimportant. This is due to the circumstance that assessments of values contributing to one’s well-being are formed at a certain point in time. Haybron and Tiberius’s (2015) approach to pragmatic subjectivism and Hall’s (2016) adaption in the context of medicine can be applied to the aim of this paper twofold: urging the integration of perspectives of affected people by assessing their values in the context of ARDs and stressing the importance of assessing lived experience by addressing potential changes in and the subjectivity of values when it comes to health-related decisions for the future. Access to individual values can be obtained, for example, by integrating empirical findings. The integration of relevant agents’ attitudes can then lead to obtaining justifiable, action-guiding recommendations for those affected and guided by accounts of well-being, specifically individuals’ own values (Haybron & Tiberius, 2015).

#### Anticipation as a framework for assessing health-related decisions

While different approaches to and components of anticipation exist in the fields of sociology, psychology, philosophy, and bioethics, my aim is to use a practice-oriented framework of anticipation for the integration of individual perspectives on advance planning in dementia research that focuses on the management of time. Adams et al. (2009) with their dimensions of anticipation compose a heuristic matrix which cannot claim to be exhaustive or applicable to all contexts, however, especially regarding their reference to examples in the field of biomedicine, I regard their matrix as well-suited for the context of anticipatory health and research decisions. Adams et al. (2009) specifically illustrate the empirical relevance of their anticipation dimensions and put forward the malleability of anticipated futures. This is why I work with their approach to reveal practical ethical dimensions of reflection on the topic of anticipation in the concrete case of ARDs. The dimensions proposed by Adams et al. (2009) may be expanded and also should, in future work, be discussed regarding their theoretical dependence. Adams et al. (2009) focus on human agency and subjective perceptions of dealing with the future and thereby identify five dimensions of anticipation, namely *injunction*, *abduction*, *optimization*, *preparedness*, and *possibility*. These dimensions and their valences are explained below. In this contribution, I use a deductive approach guided by Adams et al.’s (2009) matrix to structure the empirical data, which can be regarded as a common approach in qualitative research. The aim of this deductive approach is to further assess the concept of anticipation in relation to future projections regarding one’s own health decisions. As mentioned above, this does not imply that other dimensions may not be relevant to decision-making in the future, however, these five dimensions work well as a framework to approach the topic of anticipating dementia trajectories and dementia research participation.

The first proposed dimension of anticipation, *injunction*, refers to the entailed moral imperative by an individual or social group to anticipate life, identity, or health at risk. This highly normative dimension calls for action and a moral culture including vigilance and being informed about a potential future in light of uncertainty. *Injunction* entails the moral ideal that everybody should assess and anticipate risks for their own well-being and organize individual and social life to manage risks.

The second, *abduction*, refers to gauging potential courses of action in light of continuous eventuality and uncertainty. Conceptions of the future are deliberated against the background of moving back and forth between contingencies of the past, the present, and the future based on empirical information and abstract thinking about the available information. This epistemic dimension focuses on scientific, cultural, or communication practices, on the *how* to anticipate in practice and how anticipation is made visible in the present.

The third, *optimization*, refers to the increasing abilities to control, cope with, and transform future outcomes. Here, *optimization* can be viewed as a moral claim of responsibility of individuals to safeguard their best possible future. However, *optimization* depends on the particular socio-cultural context and constitutes a normative, social dimension that is imposed on the individual.

The fourth, *preparedness*, refers to actions of being ready for certain events as if they were already here and can be considered highly speculative and reactive. This social and normative dimension can be applied to areas of biomedical developments such as cryotechnologies for sperm or egg freezing, preparing oneself for potentially delayed family planning (Rimon-Zarfaty & Schweda, 2018; see also Rimon-Zarfaty & Schicktanz, 2022).

The fifth, *possibility*, refers to the social space of present opportunities and the options of reshaping future possibilities for what was previously unimaginable. This reflects the epistemic dimension of thinking in far-reaching ideals, often promised by new technologies. Possibilities link technological, economic, and societal values by drawing rosy prospects for society.

According to Adams et al. (2009), the result of anticipation processes is a form of conceptualizing or erasing existent problems. Here, anticipation functions as a sense of appeasing us in that “things could be (all) right if only we anticipate them properly” (Adams et al., 2009, p. 259) while at the same time stressing that the future is inevitable. This notion invokes the need to engage and act appropriately. Further, anticipation is considered a means to navigate through everyday life and maintain relations with the future. ARDs lend themselves well as a tool to concretizing the dimensions of anticipation presented above as well as to discussing further specifications of dementia in the context of dementia and dementia research.

### Historical and current aspects of advance planning and dementia research

Dementia describes a progressive neurodegenerative syndrome that affects memory, language as well as behavior and leads to affected individuals needing assistance in activities of everyday life. Alzheimer’s disease is the most common cause of dementia (Scheltens et al., 2021). Dementia is considered one of the most feared health conditions in the fourth age, as people refer to the fear of losing agency or as Gilleard and Higgs (2014) call it “ageing without agency” (p. 242). Regarding the fear of dementia, the authors differentiate between the fear of losing one’s mind and the fear of losing one’s place referring to loss of one’s status and becoming dependent (Gilleard & Higgs, 2014). A common narrative regarding the future is that the prevalence of dementia will increase continuously in industrialized countries, mainly because of increasing life expectancy. According to current estimates, around 55 million people have dementia worldwide and researchers predict this number to increase to approx. 139 million by 2050 (WHO, 2022; Scheltens et al., 2021). Hence, dementia health policy is highly framed by such future scenarios, often with negative terms such as ‘tsunami’, ‘epidemic’, or ‘threat’ (Whitehouse, 2019; Schicktanz, 2017).

Throughout history and varying across cultures, socio-cultural and political contexts have shaped how individuals perceive dementia, its symptoms and the anticipated course of the condition (Bosco et al., 2019). Historically, people affected by dementia, as with other mental illnesses, were often socially condemned or were made responsible for their condition, isolated, maltreated, or even killed (Bosco et al., 2019; Yang et al., 2016; Cohen, 1998). In contrast, some authors point out that people with early signs of dementia, at least in the past, were not pathologized as they are today, and forgetfulness was seen as a normal part of aging (Ballenger, 2017; George et al., 2016).

The origin of the word dementia derives from the Latin word *demens* (*without mind*) and its first use is accredited to the Ancient Greeks. The term “dementia” was historically used to describe different forms of intellectual deficit before being classified as a mental disorder mainly affecting aged individuals (Cipriani et al., 2011). Dementia is caused by several underlying diseases that are poorly understood. While biomedical research has focused for a long time on developing an effective treatment for later stages, the condition is still largely untreatable. Current dementia research therefore covers a broad spectrum: It targets early detection via so-called biomarkers, explores prevention strategies, and expands its scope to develop assistive technologies for living with dementia at various stages. Hence, research participation becomes relevant for a variety of stages and multiple contexts. Although nowadays dementia is classified as the result of a brain disease, it remains a highly stigmatized health condition (Yang et al., 2016; Werner, 2014; Riley et al., 2014). It is associated with psychological burdens on those directly affected, the need for care, and societal stigma as well as with an immense financial burden (Higgs & Gilleard, 2017; Prince et al., 2015). Dementia is today a paradigmatic example for ‘planning ahead’ as various studies have shown, in that individuals and their families who receive a diagnosis of early dementia feel the strong need to make plans regarding future medical care, financial issues, one’s housing situation, or even suicide (Lohmeyer et al., 2020).

The idea of planning ahead in the context of medical decision-making has existed for a long time; legal tools of healthcare advance care planning have been actively promoted since the mid-1970s in the form of living wills (Sabatino, 2010). To address shortcomings of rather static forms of documentation, the concept of advance care planning (ACP) was introduced in the 1990s. This approach can be considered a dynamic, iterative process of communication over time that aims to assess and document an individual’s values, preferences, and priorities regarding future medical treatment and care as well as to engage proxies who may also participate in future healthcare decision-making (Fleuren et al., 2020; Bronner et al., 2020; Sabatino, 2010). With ACP the underlying goals are to respect individual patient autonomy, improve quality of care, strengthen relationships in the care context including family and healthcare providers, and prepare for end-of-life (Voß & Kruse, 2019; Bosisio et al., 2018). Although ACP is considered a sensible tool also for people with dementia, to date, no established ACP-program exists for individuals in early stages of dementia (Bronner et al., 2020). Another tool for advance planning in the medical and research context that has, in some countries, been adapted for individuals in early stages of dementia is the concept of advance research directives (ARDs). ARDs are considered legal documents allowing individuals with decisional capacity to express their preferences regarding participation in future research studies for the event of cognitive incapacity (Jongsma et al., 2020; Ries et al., 2020; Andorno et al., 2016; Jongsma & van de Vathorst, 2015; Pierce, 2010).

The difference between the concepts of ACP and ARDs is that ACP can be regarded as a broader tool addressing multiple aspects of medical treatment and care including refusal of medical interventions. Instead, an ARD documents the willingness or objection to future research participation and specifies the desired type of research where applicable. While decisions regarding medical treatment and care may become necessary and inevitable, participation in research is always a voluntary option. It has previously been suggested that ARDs could be integrated into the process of ACP (Karlawish et al., 2002). Such measures can be considered valuable for a multitude of diseases. However, for a condition like dementia, due to its slow progression and its irreversibility and also due to the lack of effective treatments, it is seen as particularly helpful (Bosisio et al., 2018; Piers et al., 2018; Levi & Green, 2010).

### Dementia research as a new challenge

For my analysis, future visions of decision-making in the context of dementia and specifically in research are of interest. Thus, for considering anticipation by means of ARDs, I take a closer look at the challenges posed by including individuals affected by dementia in research settings. In the context of research, a person’s autonomy, best interest, and informed consent are generally regarded as particularly crucial. Here, prevention of any form of abuse, persuasion, or coercion are of utmost importance and can be regarded as the guiding principles in medical research ethics (Belmont Report, 1979). Further, research participants should be assured that withdrawal from research is always an option without any repercussions. The high ethical standards of research participation today can be attributed to numerous violations in the past and in Germany, specifically, to the crimes committed by physicians in the form of human experiments, coercive euthanasia, and forced sterilization of psychiatric patients during the Nazi regime as documented in the Nuremberg Trials (Roelcke, 2004; Helmchen & Lauter, 1995). Hence, the debate on ARDs is not trivial, as it touches upon aspects of autonomous health decisions and anticipating the loss thereof.

The possibility of research participation becomes more complex in the context of research with people affected by dementia or other forms of cognitive impairment. Inclusion of research participants confronts researchers with the fundamental medical-ethical issues of protecting vulnerable populations. Public discussion and historical reappraisal of the horrific and traumatic events during the Nazi era, in Germany, led to a research-critical or research-rejecting attitude and the development of critical ethical guidelines (Helmchen & Lauter, 1995). Consequently, until the end of 2016, research with people who are unable to provide consent, was rigorously restricted in Germany. Research with sole group benefit was prohibited, i.e. only research with an own benefit and minimal risk and minimal burden was allowed, to which a proxy had to consent (Marckmann & Pollmächer, 2017; Helmchen, 2015). The major barrier to including people affected by dementia in research is the uncertain diagnosis and trajectory, continuous cognitive decline, and the result that affected people are not considered fully capable (Buller, 2015; Jongsma & van de Vathorst, 2015; Helmchen & Lauter, 1995). Here, the concept of protection becomes the most crucial element.

With the changes of the Medical Products Act in 2016 adapting the regulations in Germany to an EU-regulation, research with adults who cannot provide consent can be conducted even with only group benefit, under the condition that an ARD was drafted while the research subject was able to provide consent. Still, the research may only entail minimal risk for the research subject and withdrawal should always be possible, for example, with mere utterances or gestures in line with the *natural will* (Deutscher Bundestag, 2016). The legislative amendment challenges the notion of categorically excluding this group from participating in important research and at the same time protecting individuals from abuse. To ensure this, alternative tools for including people in research and alternative consent options are being considered in the research community.

ARDs are considered such an alternative tool with the potential of respecting and safeguarding patients’ values, preferences, and decisions for research in the future in an ethically sound manner (Jongsma et al., 2020; Jongsma & van de Vathorst, 2015). The previous debate on ARDs has mainly focused on ethical acceptability under the aspect of the instability of an individual’s preferences and values, while empirical findings have shown a growing interest in ARDs among affected people for allowing them to make autonomous decisions (Jongsma et al., 2020; Bravo et al., 2011; Heinrichs, 2021). The previous debate on ARDs has, however, to a lesser extent focused on what is required by individuals in terms of anticipation for drafting such documents. With the recent changes in German legislation, such directives will become a necessity for patients to take part in non-therapeutic intervention research (Deutscher Bundestag, 2016; Haupt et al., 2018; Marckmann & Pollmächer, 2017). However, this requires efforts on a practical level to ensure that affected people are aware of such directives and receive the support and information needed (Jongsma et al., 2020). It also requires efforts on a conceptual level, namely, an examination of anticipation in the context of drafting an ARD and what implications such anticipation may have on the ethics of ARDs. Voß and Kruse (2019) have criticized in this context that existing ACP documents only refer to dementia as a future situation necessitating anticipation, thus not conceptualized for people already affected. This leads to the basic question of what I understand by the concept of anticipation and which epistemic, social, and normative challenges it may pose.

This Background section has introduced the concept of anticipation, specifically highlighting the five key dimensions of anticipation identified by Adams et al. (2009). It also provides the context for a better understanding of how dementia, related research as well as advance care planning are culturally understood by pointing to historical and conceptual debates.

## Main section: empirically informed analysis of anticipation in practice

In the following section, anticipation will be considered more closely in the context of dementia and dementia research. This includes considering the challenges dementia may pose regarding potentially changing perceptions of temporality and perceptions of research participation. The wish for planning ahead while one is still cognitively capable can refer to a multitude of life decisions or thought processes. The long-term prospect of cognitive decline may, for example, motivate decisions regarding provision of care, preventative measures, or also the wish for euthanasia. The aim of this section is to reach a more detailed understanding of anticipation in the context of ARDs by integrating theoretical and empirical material. I do this with an exploratory approach by deductively structuring the interview material with previously identified dimensions of anticipation and combining the deductive approach with an inductive analysis of the empirical material. The inductive approach is guided by a coding scheme structuring the material with a focus on attitudes and positioning towards the future which I was able to further differentiate in this secondary analysis of the data. This serves the purpose of better understanding underlying values or motivations affecting projections of the future and concrete needs to attain individually desired outcomes in the context of dementia and dementia research. The inductive approach specifically assists in deriving underlying concepts, themes, or patterns of future perspectives that are not covered with the deductive approach (Bingham & Witkowsky, 2022; Boyatzis, 1998), namely, the anticipation dimensions proposed by Adams et al. (2009). With this combined, exploratory approach, other relevant dimensions of anticipation or limitations of anticipation can be identified in the empirical material, including, for example, forms of not anticipating. With the integration of established theoretical assumptions and the empirical material, I examine experiences of the lifeworld, in the sense of lived experience, which can reveal relevant structures, meanings, and values of individuals (Rich et al., 2013).

### A detailed analysis of anticipation in dementia healthcare and dementia research

As introduced above, I here refer to Adams et al.’s (2009) dimensions of anticipation. The process of anticipation involves the conceptual separation of the past, the present, and the future, as well as individual social constructions of dementia. This may entail the separation of personal experiences regarding dementia and one’s own reflection on these experiences, separation of one’s actual values, needs, preferences, and concerns as well as current well-being, and separation of expectations or predictions of one’s potential dementia trajectory as well as engaging with changes regarding one’s values, preferences, needs, and concerns regarding care or research participation. According to Adams et al. (2009), preparedness is highly possible and endlessly malleable under the condition that individuals have a functioning conception and model of an anticipated future. Chiffi et al. (2020) further stress that anticipation comprises two distinct parts, a forward-looking attitude relating to mind and consciousness as well as active engagement in decision-making processes. Malleability, a forward-looking attitude as well as the willingness to engage, however, may become impeded with increased loss of cognitive functioning in the course of dementia. Anticipation as a concept relying on action, reflection, and thus cognitive functioning can be regarded as highly questionable in the context of dementia, which entails not only increased forgetfulness but especially decreasing abilities of planning behavior and anticipation. In that sense, dementia may encourage affected people to make timely decisions and use the window of decisional capacity to secure future events in line with one’s own preferences and values. However, the perceived narrow window of time between first perceived cognitive impairment and further decline may also put affected people under pressure to make necessary arrangements in good time. How affected people are willing to engage with anticipation, especially in the context of dementia research, will be further explored in the following sections.

### Perception, time, and liminality in the context of dementia

On the one hand, one could argue that dementia with a slowly progressing, gradual loss of decision-making capabilities, makes anticipation possible in the first place as opposed to an abrupt onset of disease, for example, in the case of cardiovascular events. Considering the sense of time, anticipatory decisions or actions could even be considered a moral imperative in the context of dementia. As known from other studies with chronic pain patients, a diagnosis often serves the purpose of providing guidance for structuring an uncertain and potentially fear-laden future (Hellström, 2001). In the context of the sociology of health and illness, e.g., Bury (1982) and Williams (2000) have examined the biographical disruption of chronic illness and the importance of timing, context, norms, expectations, and the transition from *normality* to illness. On the other hand, the socio-cultural construction of dementia, focusing on deficits and stigma, may highly affect one’s willingness to engage with an unwanted future. It may exacerbate the associated difficulties regarding communication about disease and death, especially when this involves anticipation (Bosisio et al., 2018; Bosco et al., 2019). As the ethicist Stephen Post puts it “[t]he person with cancer will retain his or her autobiography, or life story, and the sense of temporal continuity between the past, the present, and the future, but the person with AD [Alzheimer’s disease] will eventually outlive much of his or her brain. The progressive destruction of the brain before the death of the body is a more vexing social, ethical, and economic issue than is death itself” (2000, p. 1). Paradoxically, the burden of dealing with difficult decisions often leads to decisions being postponed until individuals have lost their ability to provide consent and then are totally dependent on others (Bronner et al., 2020).

As noted above, dementia is considered a chronic syndrome, associated with notions of continuum and prolonged time. As opposed to other illnesses marked by a diagnosis or a tragic event, dementia may entail an initial incident followed by phases of stability, as it does not have a straightforward linear progression (Sikes & Hall, 2018). The concept of liminality which incorporates the process of transitioning from one state to another, as in transitioning between different stages of life, can be useful in the context of dementia as Birt et al. (2017) suggest. Both subjective and social conceptions of dementia are highly relevant for the anticipation of future disease as well as advance planning in research. The loss of agency and autonomy occurs gradually over time. The creation of liminality, a transitional state, may relate to the gradual loss of one’s place in society as well as the gradual loss of cognitive abilities. One form of social engagement for people with cognitive impairment has been indicated by encouraging individuals to engage in research participation (Ries et al., 2017; Bosco et al., 2019).

While symptoms of dementia can be apparent for some time, people will perceive their “previously-envisioned selves” in a different manner (Birt et al., 2017). They further refer to the ongoing liminality dementia brings about with continuous transitions and changing demands, triggering uncertainty among those directly affected but also among caregivers. For dementia, a clearly structured *post-liminal state*, in which a perceived transition has taken place, is often not conceivable. Birt et al. (2017) emphasize the importance of overcoming states of liminality. This could include being overt about a dementia diagnosis and potentially taking control of social interactions by talking about challenges faced and by structuring future communication along individual needs. This characteristic contributes to the unpredictability of the condition, rendering planning difficult. Also, in a previous empirical study conducted by colleagues and myself (Jongsma et al., 2020), we observed such perceptions when interviewing people with subjective or mild cognitive impairment (SCI and MCI, respectively). The following quotation by an 80-year-old man with MCI illustrates the difficulty of planning for the future in light of perceived uncertainty of what to expect as well as ongoing continuity of the condition:

*[...] but you do not know how it will continue, you know* (Person with MCI, male, 80 years old, 00:28:35 − 1)

Feelings of uncertainty regarding predictability of the future and lack of control over a health condition’s progression may lead to the feeling that there is nothing one can do except wait. However, epistemic uncertainty, the wish to overcome states of liminality, or also perceived solidarity with other individuals who might find themselves in a similar state of uncertainty and liminality in the future, can potentially encourage affected individuals to engage in advance care and research planning.

### Relating anticipation concretely to advance planning in dementia research

As put forward above, not only illnesses are socially and culturally constructed, also forms of anticipation are ascribed with cultural and social norms. People experiencing a condition or individuals witnessing a loved one’s experience are likely to apply an interpretation to the established conception based on their own understanding of dementia (Bosco et al., 2019). This shifting conception of the health condition is likely to affect their willingness to engage with anticipation. However, previous studies have shown that the interest and willingness to engage in social and medical decision-making continues in early stages of dementia (Bronner et al., 2020; Jongsma et al., 2020).

In the following, I use a deductive approach by referring to those key dimensions by Adams et al. (2009) (see above) relevant to the assessment of the empirical data. These dimensions and their valences will be used to guide the further analysis, while abstracting further dimensions that may become relevant with an inductive approach to derive underlying concepts, themes, or patterns of future perspectives which may not be covered with the deductive approach.

#### Injunction and preparedness

While much research has focused on the unpredictability of the condition and the instability of an individual’s preferences and values, limiting planning behaviors, the aspect of time, in turn, may also afford space for planning and strategies for coping with or viewing the future. Early decisions regarding research participation, before advanced cognitive decline, could lead to a feeling of retaining control over one’s own body and health decisions. According to Adams et al.’s (2009) dimensions of anticipation; *injunction* refers to a moral plea to prepare and act in order to maintain one’s autonomy and individual preferences. *Preparedness* means that individuals want to plan ahead and be ready for inevitable future incapacity. Both forms of anticipation emerged strongly in interviews with persons in prodromal stages of dementia. Such reactions of maintaining autonomy to make health decisions over time are poignantly illustrated in the following statements of interviewed people at varying ages assessing the benefits of preparing and planning ahead. A 70-year-old man with SCI stated:

*It is better if the one affected arranges that in advance, of course. […] Yes, it’s a matter of autonomy. I mean, humans as such like to be autonomous and independent, I assume that the majority of people prefers to be independent than dependent. […] Yes, I assume that a person wants to be autonomous, likes to decide independently as long as he or she can. I cannot imagine that one would say, yes, I prefer to let others decide for me, maybe there are people like that but I can’t imagine that* (Person with SCI, male, 70 years old, 00:19:54 − 7)

While, in a similar vein, a 56-year-old woman diagnosed with MCI stated:

*I would prefer if I could decide that myself now. […] Yes, because then I decided it, because I still know what I want now. And for me something like that is very important to me* (Person with MCI, female, 56 years old, 00:21:32 − 6)

The notion of *preparedness* is further stressed by the insight of affected persons that the condition progresses slowly and detailed information can be assessed in advance:

*I think it’s very good [that] there are many other things that dementia patients can consciously decide on before the symptoms get worse and I find it good then also that one […] when you get the diagnosis that is often still a stage in which you can make such decisions that you can sign an informed consent form for research* (Person with SCI, female, 47 years old, 00:08:59 − 2)

These conceptions relate to the fact that an individual is the only one who knows their own preferences and values and that it is part of human nature to make such decisions independently and autonomously. The dimensions of anticipation – *injunction* and *preparedness* – apply here based on the assumption that values, preferences, and belief systems are likely to remain stable in the course of a health condition.

#### Optimization

As in other forms of advance planning in healthcare and specifically in the context of dementia, the notion of becoming a burden plays an important role in the willingness to anticipate. Making provisions and decisions for future care, and, specifically, research, is often regarded as a form of relieving perceived burden from family members or also from oneself (Jongsma et al., 2020; Bally et al., 2020). The sentiment of perceived relief for one’s own behalf or for the family by making decisions in advance is precisely illustrated in the following statements by two affected persons:

*Yes, I would […] so that afterwards no one else has to think about it, so that my wife does not have to decide or the children have to decide if the question then comes to them we can do it with your dad or with your husband, then my wife or whoever can say here my [husband or] dad has decided that and that is good for me that you still decide, that is how it has to be done* (Person with SCI, male, 78 years old, 00:29:15 − 3)

*In any case, I think that this is a relief because at the time when you can still decide it is sensible because then you have it for later then. In that sense, I find that very useful* (Person with SCI, female, 45 years old, 00:20:25 − 5)

This conception of relieving burden can be connected to Adams et al.’s (2009) anticipation dimension of *optimization. Optimization* here means that responsibility is taken on to secure the best possible future for oneself by making decisions in advance, so that others do not have to make them for you. The aspect of time plays an important role, making decisions while one is still capable, for the future in which one may not be anymore.

#### Possibility

Decisions made in the context of research and research participation may be considered less existential than other healthcare decisions. However, especially in the context of dementia, where currently no cure exists, the concept of research and the necessity to involve people with dementia in research is framed by affected people as well as by researchers in a mainly positive manner (Jongsma et al., 2020; Ries et al., 2020). The underlying rhetoric, also among affected people, seems to be guided by the perceived importance of research to better understand the condition’s etiology and that, although it may take many years, it is important to involve affected people in research to possibly change treatment options in the future, as illustrated in the following statement:

*I think it’s important, maybe someday you’ll find something so you can get to the bottom of it that maybe you can turn it off somewhere. So that it takes many years and decades for such things that seems plausible to me, but I guess […], where can you get the knowledge if not from such studies, the knowledge has to come from somewhere* (Person with MCI, female, 56 years old, 00:18:11 − 0)

The perceived importance and willingness to engage in research can be linked to Adams et al.’s (2009) anticipation dimension of *possibility* in that new forms of opportunities are made possible. These empirical findings, as well as other empirical research, frame willingness and appropriateness of engaging in research highly contingent on the progression of symptoms of dementia as well as on the kind of research (Jongsma et al., 2020; Ries et al., 2020). Motivations for participation in research range from the desire to promote medical innovation to acting in terms of solidarity for future benefit, as this following statement illustrates:

*For me [research] is just interesting, […], also if it does not help me now, if it will help future generations or others, then why not participate* (Person with SCI, female, 44 years old, 00:32:58 − 5)

Some forms of motivation also derive from the faint hope that participants would potentially benefit from research themselves:

*I’d say first and foremost, I’d like to benefit myself, but I think it can’t be wrong if it’s also helpful for other people* (Person with SCI, female, 45 years old, 00:19:30 − 8)

Regarding the concept and use of ARDs, the dimension of *possibility* becomes especially important: Research with vulnerable groups is newly reconfigured in light of the urgent sense that new treatments are on their way, promoting the necessity to engage in research studies. Therefore trust in researchers can be considered an important factor put forward by affected people, based on knowledge about abuse in history and on decreasing decisional capacity in the course of dementia (Jongsma et al., 2020).

#### Limitations of anticipation: proxy decision-making and fatalism

Despite high willingness to participate in research and the perceived importance of making autonomous decisions in advance with the drafting of an ARD, difficulties remain in exploring and being able to anticipate how one would feel in more advanced stages of dementia. In early stages of dementia, an ARD thus is regarded by some as a very useful tool to preserve self-determined decisions. But also fear exists of not remembering that one had signed such a document. This makes anticipated decisions for the future extremely difficult and creates uncertainty about how well one knows oneself and how predictable one’s own but also others’ decisions are. People seem aware that anticipation means that one does not fully know what is coming, but preexisting knowledge shapes what would be preferable or what could happen. In the context of ARDs, a trusted person, a proxy or family member, is considered a possibility to ensure that potentially incorrect anticipation or the potential lack of anticipatory decisions can be dealt with, as this woman with SCI stated:

*[…] so I think a trusted person, so in addition, I have someone who decides according to my wishes. I find that quite sensible because of the open question how my dementia will develop […] to ensure that I have options to withdraw and I still will be taken seriously* (Person with SCI, female, 44 years old, 00:12:08–8)

Other forms of advance planning have for a long time stressed the importance of including proxy decision-making as a supplementary support system for directives and framing the advance planning process as an iterative and dynamic one (Heinrichs, 2021; Fleuren et al., 2020; Bronner et al., 2020; Voß & Kruse, 2019; Sabatino, 2010). The difficulty to anticipate in the context of dementia, as shown above, is conceptualized in what individuals deem feasible at a certain point of time and stage of dementia which may change in the course of its progression. Anticipation in the context of a chronic condition such as dementia is increasingly correlated with individuals facing uncertainty. At the same time, individuals may face an appeal for being prepared to ensure self-determined decisions throughout the course of dementia, for example, by including a proxy, prompted by the moral necessity to be ready for future events.

My analysis shows that, in the context of dementia, anticipation is mainly encouraged by a wish to make self-determined and independent decisions (or also not to plan) before further cognitive decline, by perceived responsibility to secure a future in line with one’s own assumed best interest or well-being, and by a perceived desideration to engage in dementia research. However, the analysis also highlights limitations of anticipation due to uncertainty posed by the dementia trajectory and the need to rely on others to potentially support them with ARDs.

According to my analysis, four of the five dimensions of anticipation as proposed by Adams et al. (2009) found resonance in conceptions regarding anticipation in the context of research participation by means of ARDs, but the dimension *abduction* did not. However, based on the inductive analysis of the empirical material, I suggest adding a further dimension regarding future projections in the context of dementia and participation in dementia research, namely, *fatalism*. *Fatalism* refers to an individual’s sense of resignation due to lack of control over a health situation. This dimension emerging in the context of perceived future incapacity due to prospective dementia, especially regarding the dementia trajectory, however, can be considered more comprehensive, as it here encompasses a spectrum ranging from utterances referring to living in the present to reactions of denial or resignation. This sentiment of not planning or living only in the moment is pointedly illustrated in the following two statements by people with SCI and one statement by a person with suspected vascular dementia:

*No, no, I do not plan anything for that. I’ll just take things as they come. In that case, I’m a historian, and then I say these are things you cannot prevent and then you have to, you have to go with it […]* (Person with SCI, male, 70 years old, 00:05:27 − 1)

*Not at the moment because you do not know [...] what’s going to happen, there I am, I always plan from one day to the next* (Person with SCI, male, 78 years old, 00:05:23 − 6)

*[Regarding future planning], well, I fear that it will get worse and worse, and […] it is better to push it away than to think about it intensely* (Person with suspected vascular dementia, male, 81 years old, 00:05:20 − 1)

On an individual or even collective level, dealing with (unpredictable) future events such as the onset of dementia can be considered in a fatalistic manner as previous research has also found (e.g., El Haj et al., 2020; Rimmer et al., 2005). As fatalistic attitudes I also understand potentially missing intentions to change behavior or situations, to accept the problems or suffering that come with the prospect or onset of a health event, often conceptualized as an external, uncontrollable catastrophe. Fatalism in the context of health can further entail, for example, not taking up preventative or even curative measures (Keeley et al., 2009). As such, fatalism can be understood as an antipode towards anticipatory attitudes, thus the active decision not to engage in anticipatory decision-making.

These perceptions mirror the social and cultural constructions of dementia, for example, the focus on deficit, and show how these perceptions affect individual willingness and the perceived necessity to engage with anticipation. For many affected people, forms of anticipation seem to be crucial for the dementia trajectory. When considering dementia trajectories, it may be helpful to more strongly consider the subjectivity of values as well as to regard time as less linear and recognize temporal diversity and different forms of temporal agency. As Lemos Dekker (2020) stresses, the present is always infused with conceptions of the past and the future, meaning that past experiences and orientations towards the future are crucial for present experiences and their individual meaning.

## Conclusions, implications for clinical practice, and future directions

In this concluding section, I will summarize and assess the conceptual and empirical findings relating to anticipation, give a constructive outlook on what implications these findings may have for clinical practice, and also point towards a future need for research.

The main theses from the deliberations about anticipation in the context of dementia and dementia research in light of future incapacity can be summarized as:

Anticipation regarding drafting an ARD is regarded as beneficial for *preparedness* in the context of dementia and advance research planning. Moreover, it becomes a moral imperative in the form of *injunction* in order to maintain one’s autonomy and individual preferences in this context. Anticipation regarding drafting an ARD is regarded as beneficial for *optimization* in that responsibility is taken to secure one’s own best possible future and to reduce reliance on and potential burdening of others. The perceived importance and willingness to engage in research functions as a form of anticipation directed at (potentially limited) *optimization* and *possibility* of new opportunities for advances in medicine. However, regarding the dementia trajectory, the imagined fear about aging concerning potential cognitive and physical impairment as part of the conceptions of the fourth age strongly influence how people affected by cognitive impairment are willing to anticipate future progression of dementia and their future envisioned selves as well as their ability to actually imagine a state in which one is more strongly cognitively impaired. Adams et al.’s (2009) anticipation dimension *abduction* was not identified in the empirical material. As elucidated above, *abduction* refers to the assessment of potential courses of action in light of ongoing eventuality and continuity, negotiating conceptions of the future based on experience made in the past. In the context of dementia and research participation, anticipation could, for example, relate to potential treatment development for diseases that cause dementia. *Abduction*, more strongly than the other dimensions presented, necessitates experience-based knowledge. As the concept of ARDs and involving affected people in research can be considered a new development, experience-based knowledge currently does not exist for the interviewees. This could explain why the interviewees did not refer to aspects of *abduction*. However, this does not exclude that *abduction* could become relevant in the future, once participation in research designs and treatment development in the context of dementia are more established.

My analysis shows that further dimensions or limitations can be identified in the context of anticipation, here including the option of proxy decision-making as a supplementary support system and fatalism. Both highly relate to the perceived dementia trajectory and include a spectrum ranging from wanting support in representing one’s own wishes, utterances referring to living in the present to reactions of denial or resignation. Other dimensions may also be relevant for advance planning in dementia and dementia research, which should be explored in future research, especially for considering concrete obstacles to sustainably implementing ARDs in clinical practice. In the context of dementia, further dimensions could, for example, entail aspects of monitoring (e.g. Lupton, 2013) in that individual capacities are observed and estimates are made considering how capacities might develop or change in the future. In addition, prevention concerning actions to potentially avoid negative future outcomes could also become relevant as an anticipation dimension.

My findings imply that motivation for and anticipation of research participation and conceptions of the dementia trajectory can be viewed as slightly separate, but closely related entities. This potentially poses difficulties for the practical implementation and counseling around ARDs. When planning ahead and trying to predict a future self, when deciding for or against participating in research in the form of drafting an ARD, anticipation can help as a concept to combine past and present experiences with projections of the future to make such decisions more tangible. The concept of imagination and the act of concretely inferring potential outcomes, preferences, values, and forms of existence feed into what anticipation entails and clearly mark the process of anticipation not only as a vague consideration but as an actual form of action. However, projecting future outcomes based on lived experience becomes extremely difficult if one has not experienced dementia and one does not know how one may feel at different stages of progression. This severely limits the scope of anticipation possible in the context of being a person affected by dementia.

Considering how Adams et al. (2009) frame anticipation, it does not necessarily require specialized knowledge or skills; it can be accomplished by lay people in a highly individualized manner. Moreover, it actually becomes an inherent aim and imperative to anticipate. However, the conception of anticipation as presented by Adams et al. (2009) relies heavily on the ability to weigh options and make projections of the future, and reconsider them over time, requiring cognitive capacities. This kind of anticipation, requiring reconsideration over time, can be regarded as highly challenging in the context of the dementia trajectory, which further strengthens the conceptual limitation I identified and described above. Hence, dementia’s inherent loss of agency and perceived decision-making capacity encourage the exploration of further relevant dimensions of or limitations to anticipation such as involving proxy decision-making or attitudes of fatalism which can be regarded as a form of resistance to anticipation. Here the question arises what implications the sentiment of potentially not wanting to anticipate has for advance planning in dementia research and specifically for the drafting and use of an ARD.

Apart from the conceptual limitation, on an individual level, the differentiation between the willingness to explore one’s future illness trajectory and the willingness to participate in research in the future assisted by ARDs seems to be of high relevance for anticipation. While the dementia trajectory is perceived as inevitable by most affected people, research participation is voluntary and leaves room for opting for or out of certain types of research. Thus, affected people may be more willing to engage with anticipation regarding research than with anticipation of the dementia trajectory itself and what it may entail on a personal level. However, as my findings show and as mentioned above, anticipation regarding research as well as regarding the dementia trajectory may be slightly separate from another but the two are highly intertwined. This means that anticipation of research participation is difficult to assess without taking the dementia trajectory into account. Moreover, planning your life around anticipatory forms of engagement has an effect on physical, mental, and emotional well-being and vice versa (Adams et al., 2009; Bosco et al., 2019). Hence, forms of anticipation regarding an illness may influence the way in which an illness is accepted or integrated into one’s life, which, in turn, may highly affect the manner in which other aspects of anticipation, such as research participation, are perceived and framed. The exploration of underlying values or motivations affecting projections of the future and the specification of concrete needs to attain desired outcomes in the context of dementia and dementia research by integrating empirical material can contribute to assessing the applicability of theoretical frameworks on anticipation. While Adams et al.’s (2009) approach can be regarded as suitable for exploring perspectives of anticipation in the context of dementia, their dimensions necessitate a conceptual expansion of not anticipating or a reluctance to anticipate. It should be further explored whether other dimensions, such as the ones referred to above, may become relevant in the broader field of future health or research decisions.

Given the potential changes an individual may face in the course of dementia, dementia research ethics especially focuses on incorporating affected individuals’ values, wishes, and needs (Robillard & Feng, 2017). The acknowledgement of potentially changing preferences and values can be regarded as very useful in the drafting of an ARD and considering forms of anticipation. Especially regarding the underlying uncertainty of such changes in the future, the voluntariness and the option of opting out of research participation must be maintained. ARDs and ACP in general can be regarded as a means to actively apply anticipation to the uncertain future; advance research planning can then be regarded as one relevant form of anticipating the future (Haupt et al., 2018; Miyata et al., 2006).

Not only is there a conceptual limitation posed by needing to project future outcomes without lived experience of dementia, the practice of anticipation in the context of dementia research, potentially initiated by physicians, researchers, or policy makers, also poses bioethical issues. For example, the question arises whether the ethical-legal justification of group benefit for research participation shifts the focus from individual trajectories to a more generalized picture. Hence, individual (preexisting) values regarding solidarity with other affected people in the form of anticipating future group identity, gratefulness to science, or preferences towards autonomy might steer the decision in favor of or also against the drafting and use of an ARD.

Thus, the process of anticipating ACP and ARDs should be considered a dynamic, iterative process of communication over time, taking into account that new or innovative forms of communication and transferring of information may become necessary for the case of changing preferences and concerns as well as changing research options. Similar approaches of ACP have been developed explicitly for the context of dementia by Bronner et al. (2020) and Voß & Kruse (2019). Bronner et al. (2020) for their decision-assisting tool focused especially on relevant topics that were deemed anticipatable and entailing good predictability, such as healthcare proxies, advance directives, living arrangements, car driving, and inheritance. A similar list of aspects relevant to ARDs could be developed that are deemed especially anticipatable by affected people and discussed routinely with individuals who have drafted such a directive. Previously, anticipation or expectations have been primarily explored in the context of early diagnosis and biomarker testing from the perspective of healthcare professionals (e.g. Swallow, 2017). Thus, the perspective of affected people needs further recognition in this context. For example, concerning the strong wish to make self-determined and independent decisions, as identified in the empirical findings, it would be important to assess in clinical practice what concrete decisions these could be and what implications they have for individual conceptions of well-being. In a counseling setting on ARDs, as in the study by Voß & Kruse (2019), such a conversation could include self-assessment of one’s own values and wishes and how these may change over time.

Further, specialized training may become relevant to engage with potentially changing values, preferences, needs, and concerns. Such an approach would allow for new entry points for anticipation of dementia and advance research planning using ARDs. Here, considering how information should be framed comprehensibly and assessing which preferences and values are individually expected to remain stable in the course of dementia need to be addressed. Further, future research should more closely explore whether the willingness to engage in social and medical decision-making is extended beyond early stages of dementia. It will also be fruitful to further explore how anticipation in the medical sphere is different from anticipation of everyday life.

Regarding the dementia trajectory, affected people may inaccurately predict future decisions due to an overestimation of the impact that specific incapacities will have on their lives, or they may misjudge the actual impact of various incapacities. Correspondingly, people may underestimate their ability to adapt to even the most severe incapacities. Further empirical research should be conducted on subjective perceptions of the dementia trajectory and to which extent perceptions of a radical or transformative experience impact anticipatory decision-making (e.g. Paul, 2014). Anticipation is not directed necessarily at a better future but rather at a future that is regarded as individually most feasible. The potential of anticipatory regret should also be considered, i.e. the potential negative consequences of a decision. Regarding ARDs it remains unclear whether the supplementary function of proxy decision-making is sufficient to counteract anticipatory regret. While anticipated regret has been explored in the context of health behavior in general (e.g. Brewer et al., 2016), and more specifically, as a motivator for acting on forms of dementia prevention (e.g. Schicktanz, 2020), regret regarding unfavorable anticipation in hindsight still requires further scientific attention.

## Data Availability

All data was available to the author at all times.

## Footnotes

1 In this qualitative study we included 24 people with subjective cognitive impairment (SCI) or mild cognitive impairment (MCI) in Germany. In semi-structured interviews we asked participants about the following topics: receiving the diagnosis, dementia research, informed consent and research participation, and their perceived use of ARDs. None of the participants had previously completed an ARD, given that the changes in German law are very recent and due to the fact that ARDs are not yet fully operational in clinical practice and no standardized template currently exists for the German context. The interviewer elucidated the concept of an ARD based on the concept of an advance care directive as a document that records patients’ preferences and decisions. Participants were recruited by various channels including patient organizations, newspaper advertisements, the university hospital, and snowball sampling to elicit a wide range of experiences with cognitive decline and types of impairments across the condition. The data was analyzed by means of thematic analysis and combined deductive and inductive coding. For further methodological details and supplemental material see Jongsma et al., 2020.

2 For further and more extensive overviews of the different strands and influences of perceptions of the future in the field of sociology, see for example: Beckert & Suckert, 2021. In this contribution, the reference to the field serves not as a comprehensive exposition but rather as a form of contextualizing anticipation in the field of health-related decision-making in and for the future.

3 The work by Adams et al. (2009) is referenced widely in the context of medical advances and medical decision-making, specifically focusing on the negotiation of potentiality and, more generally, anticipatory biomedicine. However, most authors use Adams et al. (2009) to frame aspects of temporality and assessments of potential action regarding the future and do not use Adams et al.’s (2009) anticipation dimensions as a heuristic matrix like I do, see for example: Kroløkke & Bach, 2020; Swallow, 2020; Kendig & Bauchspies, 2021; Bogicevic & Svendsen, 2021.
